# A high‐fat diet supplemented with medium‐chain triglycerides ameliorates hepatic steatosis by reducing ceramide and diacylglycerol accumulation in mice

**DOI:** 10.1113/EP091545

**Published:** 2024-01-09

**Authors:** Stephanie Mourad, Abdualrahman Mohammed Abdualkader, Xiaobei Li, Shailee Jani, Rolando B. Ceddia, Rami Al Batran

**Affiliations:** ^1^ Faculty of Pharmacy Université de Montréal Montréal Quebec Canada; ^2^ Montreal Diabetes Research Center Montréal Quebec Canada; ^3^ Cardiometabolic Health, Diabetes and Obesity Research Network Montréal Quebec Canada; ^4^ Muscle Health Research Center, School of Kinesiology and Health Science York University North York Ontario Canada

**Keywords:** ceramide, diacylglycerol, hepatic steatosis, inflammation, medium‐chain triglycerides

## Abstract

Non‐alcoholic fatty liver disease (NAFLD) is projected to be the most common chronic liver disease worldwide and is closely linked to obesity, insulin resistance and type 2 diabetes. Currently, no pharmacological treatments are available to treat NAFLD, and lifestyle modification, including dietary interventions, is the only remedy. Therefore, we conducted a study to determine whether supplementation with medium‐chain triglycerides (MCTs), containing a mixture of C8 and C10 (60/40), attenuates NAFLD in obese and insulin‐resistant mice. To achieve that, we fed C57BL/6 male mice a high‐fat diet (HFD) for 12 weeks to induce obesity and hepatic steatosis, after which obese mice were assigned randomly either to remain on the HFD or to transition to an HFD supplemented with MCTs (HFD + MCTs) or a low‐fat diet (LFD) for 6 weeks as another dietary intervention model. Another group of mice was kept on an LFD throughout the study and used as a lean control group. Obese mice that transitioned to HFD + MCTs exhibited improvement in glucose and insulin tolerance tests, and the latter improvement was independent of changes in adiposity when compared with HFD‐fed mice. Additionally, supplementation with MCTs significantly reduced hepatic steatosis, improved liver enzymes and decreased hepatic expression of inflammation‐related genes to levels similar to those observed in obese mice transitioned to an LFD. Importantly, HFD + MCTs markedly lowered hepatic ceramide and diacylglycerol content and prevented protein kinase C‐ε translocation to the plasma membrane. Our study demonstrated that supplementation with MCTs formulated mainly from C8 and C10 effectively ameliorated NAFLD in obese mice.

## Abstract (highlights)

- **What is the central question of this study?**
  Medium‐chain triglycerides (MCTs) originate from coconut oil. Variations in the types of MCT molecules and their ratios influence their hepatoprotective properties. We aimed to investigate the effects of supplementation with MCTs, consisting primarily of C8 and C10, and not C12 that is abundant in coconut oil, on hepatic steatosis.
- **What is the main finding and its importance?**
  Supplementation with MCTs reduced hepatic steatosis and inflammation while improving liver enzyme levels in obese mice. Moreover, supplementation with MCTs lowered hepatic lipotoxicity and enhanced glucose homeostasis independent of adiposity.

## 1 INTRODUCTION

Non‐alcoholic fatty liver disease (NAFLD) is a progressive liver disease that affects ∼25% of the global population (Younossi, Golabi, et al., 2023). The prevalence of NAFLD has increased significantly along with the global rise in obesity and type 2 diabetes, which are well‐known risk factors for NAFLD (Loomba et al., 2021). Non‐alcoholic fatty liver disease is characterized by excessive intrahepatic triglyceride accumulation without excess alcohol consumption (Hodson & Gunn, 2019) and encompasses a spectrum of well‐defined liver pathologies ranging from simple hepatic steatosis to non‐alcoholic steatohepatitis (NASH) (Michelotti et al., 2013). Non‐alcoholic steatohepatitis can progress to end‐stage liver diseases, such as cirrhosis and hepatocellular carcinoma, by activating inflammatory cascades and fibrogenesis (Anstee et al., 2019). Despite these numerous adverse outcomes, there are no Food and Drug Administration‐approved therapies specifically to treat NAFLD, and lifestyle modification, including dietary interventions, currently remains the mainstay of NAFLD (Younossi, Zelber‐Sagi, et al., 2023).

Medium‐chain triglycerides (MCTs) are categorized as medium‐chain fatty acids (MCFAs) that can be used as a non‐pharmacological treatment for various neurological and metabolic disorders, including epilepsy, Alzheimer's disease and insulin resistance (Borges et al., 2019; Geng et al., 2016; Ota et al., 2019). Unlike long‐chain fatty acids that come from long‐chain triglycerides, MCFAs released from MCTs are transported through the portal vein directly to the liver (Lemarié et al., 2016), where they are metabolized rapidly through β‐oxidation because they do not require the carnitine shuttle system (Bach & Babayan, 1982). This distinct metabolic property also makes MCTs an attractive remedy for long‐chain fatty acid oxidation disorders (Gillingham et al., 2017). Medium‐chain triglycerides are a form of saturated fatty acid with a chain length ranging from C8 to C12 and are found naturally in coconut and palm oils (Babayan, 1968). Although MCTs are produced from coconut and palm oils through a process called fractionation, MCTs and coconut oils have several differences, namely the proportions and types of MCT molecules that they contain (Clegg, 2017; Denke & Grundy, 1992). Coconut oils typically contain 50% C12 (lauric acid) and minor quantities of C8 (caprylic acid) and C10 (caproic acid) (Denke & Grundy, 1992), whereas MCTs are made either as pure C8 or a mixture of C8 (50%–80%) and C10 (20%–50%) (Bach & Babayan, 1982).

Owing to variations in the ratios and types of MCT molecules found in nutritional products with MCTs, a controversy has emerged regarding their hepatoprotective effects. For instance, some studies have shown that virgin coconut oils, containing high amounts of C12 and moderate quantities of C8 and C10, exacerbate hepatic steatosis in animals (Piot et al., 1999; Ströher et al., 2020), whereas others have found the opposite outcome (Narayanankutty et al., 2018; Panchal et al., 2017). To explore the potential hepatoprotection of specific MCT molecules, namely C8 and C10, we fed obese mice a high‐fat diet (HFD) supplemented with a mixture of MCTs (60% C8 and 40% C10) for 6 weeks. Our findings demonstrate that this supplementation with MCTs effectively reduces hepatic steatosis and inflammation while enhancing liver enzyme levels in obese mice. Additionally, supplementation with MCTs lowers hepatic ceramide and diacylglycerol content and prevents protein kinase C‐ε (PKCε) translocation to the plasma membrane. These results highlight the potential benefits of incorporating a balanced blend of C8 and C10 MCTs in dietary supplementation for liver health.

## 2 MATERIALS AND METHODS

### 2.1 Ethical approval

Animal experiments were approved by the Animal Experimentation Ethics Committees of the Université de Montréal (approval number 20‐062) and performed in accordance with the Guide for the Canadian Council on Animal Care.

### 2.2 Animal care and dietary composition

Animals were housed in a 22°C temperature‐controlled unit under a 12 h–12 h light–dark cycle with standard environmental enrichment and ad libitum access to drinking water and food. Seven‐week‐old male C57BL/6J mice (The Jackson Laboratory) were fed either a low‐fat diet (LFD; 10% kcal from lard; Research Diets D12450J) and used as the lean control group for comparison or an HFD (60% kcal from lard; Research Diets D12492) for 12 weeks to induce obesity, insulin resistance and hepatic steatosis. After 12 weeks of the dietary protocol, mice were randomized into three dietary intervention groups for 6 weeks. Mice were maintained on the HFD or switched to an HFD containing 15% w/w MCT oils (HFD + MCTs; 30% kcal from MCT oils; Research Diets D22012006) or switched to an LFD. The detailed composition of the three diets is indicated in Table 1. The proportion of calories derived from protein (20%) and the sucrose content (5%) remained consistent across all the diets. Hence, the diets varied solely in terms of the relative abundance of complex carbohydrates and sources of fats. The HFD contained 60% of its calories from fat and 20% from carbohydrates. In contrast, the HFD + MCTs contained 60% of its calories from fat, with 50% of the fat content derived from MCT oils (60% C8 and 40% C10), along with 20% from carbohydrates. Lastly, the LFD contained 10% of its calories from fat and 70% from carbohydrates. The macronutrient sources remained the same for all diets, including the protein source (casein and l‐cystine) and the carbohydrate source (corn starch, maltodextrin 10 and sucrose). During the 6 week dietary intervention, body weight and the circulating glucose and ketone body levels were measured weekly. Six weeks after commencing the dietary intervention, mice were killed during the fed state using an i.p. injection of sodium pentobarbital (12 mg), after which tissues were extracted and immediately snap frozen in liquid N_2_ via liquid N_2_‐cooled Wollenberger tongs.

**TABLE 1:** Composition of diets.

	Low‐fat diet		High‐fat diet		MCTs diet	
Ingredient	(g)	(kcal)	(g)	(kcal)	(g)	(kcal)
Casein	200	800	200	800	200	800
l‐Cystine	3	12	3	12	3	12
Corn starch	506.2	2024.8	0	0	0	0
Maltodextrin 10	125	500	125	500	125	500
Sucrose	68.8	275.2	68.8	275.2	68.8	275.2
Cellulose, BW200	50	0	50	0	50	0
Soybean oil	25	225	25	225	25	225
Lard	20	180	245	2205	129	1161
MCT oils^a^	0	0	0	0	116	1044
Mineral mix, S10026	10	0	10	0	10	0
Dicalcium phosphate	13	0	13	0	13	0
Calcium carbonate	5.5	0	5.5	0	5.5	0
Potassium citrate.1 H_2_O	16.5	0	16.5	0	16.5	0
Vitamin mix, V10001	10	40	10	40	10	40
Choline bitartrate	2	0	2	0	2	0

^a^ 60% C8 and 40% C10.

Abbreviation: MCT, medium‐chain triglyceride.

### 2.3 Glucose, insulin and pyruvate tolerance tests

Glucose tolerance tests (GTTs) and insulin tolerance tests (ITTs) were performed in mice fasted overnight (15 h) or for 6 h, respectively. Mice were injected i.p. with 2 g/kg of glucose or with 0.5 U/kg of insulin, respectively. For pyruvate tolerance tests (PTTs), mice were fasted overnight to activate gluconeogenesis and were then injected with 1.5 g/kg of sodium pyruvate. For all tests, blood glucose concentrations were measured from tail whole‐blood following the fast (0 min) and at 15, 30, 60, 90 and 120 min postinjection using the Contour Next blood glucose monitoring system (Bayer). For the assessment of circulating insulin levels, plasma was collected during the GTT at 0 and 30 min after the glucose injection and analysed using a commercially available enzyme‐linked immunosorbent assay kit according to the manufacturer's instructions (Alpco Diagnostics) and as described previously (Al Batran et al., 2020).

### 2.4 Assessment of ketosis

Circulating concentrations of the ketone body β‐hydroxybutyrate (βOHB) were measured from tail whole blood during the fed and fasting states using FreeStyle Precision Blood β‐Ketone (Abbot Diabetes Care) as previously described (Mechchate et al., 2023).

### 2.5 In vivo metabolic assessment

Indirect calorimetry assessment was performed using the Comprehensive Laboratory Animal Monitoring System (CLAMS; Columbus Instruments) for in vivo metabolic monitoring. The experiment was conducted after an initial 24 h acclimation period, and whole‐body oxygen consumption, carbon dioxide production, respiratory exchange ratios, energy expenditure, food intake and physical activity data were collected every 6 min and quantified for 48 h in animals with free access to food and drinking water. Hourly outputs from the CLAMS files were generated utilizing the Web‐based CalR tool (Mina et al., 2018).

### 2.6 Biochemical analysis

Immediately post mortem, blood was collected by cardiac puncture and centrifuged at 18,928 *g* for 10 min at 4°C. The plasma was collected and kept at 80°C for further analysis. Plasma triglycerides (FUJIFILM Wako Diagnostics, catalogue numbers 994‐02891, 990‐02991, 464‐01601 and 416‐00102), cholesterol (FUJIFILM Wako Diagnostics, catalogue numbers 999‐02601 and 416‐00102), non‐esterified fatty acids (FUJIFILM Wako Diagnostics, catalogue numbers 999‐34691, 995‐34791, 991‐34891, 993‐35191 and 276‐76491), alanine transaminase (Sigma, catalogue number MAK052) and aspartate transaminase (Sigma, catalogue number MAK055) levels were measured according to the kit protocols.

### 2.7 Histological analysis

Post mortem, a section of the liver from all experimental groups was collected and fixed in 10% formalin. Subsequently, 4‐μm‐thick paraffin‐embedded liver sections were stained with Haematoxylin and Eosin to visualize the pattern of lipid accumulation at the Institute for Research in Immunology and Cancer, Histology Core Facility. Slide images were captured using a NanoZoomer 2.0‐HT digital slide scanner (Hamamatsu), and quantification of liver fat content was computed in a blinded fashion using ImageJ software, followed by averaging the percentage area of fat droplets for 20 randomly selected fields at ×40 magnification. The NAFLD activity score (NAS) was calculated according to the NASH Clinical Research Network scoring system (Kleiner et al., 2005; Younossi et al., 2018). Briefly, the NAS (0–8) was determined by the sum of scores of steatosis (0–3), lobular inflammation (0–3) and hepatocyte ballooning (0–2).

### 2.8 Assessment of hepatic triglycerides and cholesterol content

Frozen liver tissues were extracted in screw tubes containing 2:1 chloroform:methanol solution and stainless‐steel beads, as previously described (Al Batran et al., 2019). Briefly, the tubes were shaken for 3 min at 25 Hz/s using a TissueLyser II (Qiagen), then put on ice for 30 min. Afterwards, the supernatant was collected, transferred into new tubes, and the lower phase was obtained by adding 0.04% CaCl_2_, followed by centrifugation at 4,032 *g* for 20 min, repeated twice. Next, methanol was added to obtain one phase, and samples were dried, re‐solubilized in isopropanol and used to measure concentrations of hepatic triglycerides and cholesterol using the kits mentioned above according to the manufacturer's instructions.

### 2.9 Assessment of hepatic diacylglycerol and ceramide content

The lipid samples extracted from liver tissue using Folch's method (Folch et al., 1957) were analysed using an ultra‐high‐pressure liquid chromatography system (UHPLC‐UV; Nexera X2, Shimadzu, Kyoto, Japan) to determine the total amounts of diacylglycerol and ceramide content. To prepare the samples for chromatographic analysis of diacylglycerol, 50 mg of liver tissue was homogenized in 200 μL of chloroform:methanol (MeOH, 2:1 v/v), dried overnight under nitrogen gas, and resuspended in 110 μL of 2‐propanol‐hexane (ProHex, 5:4 v/v). Quantification was carried out using the UHPLC‐UV detection machine, as previously described (Jani et al., 2022). Samples were automatically injected into a reverse‐phase column (C18 5 μm, 250 mm × 4.6 mm), and the chromatography was conducted using a gradient of MeOH and ProHex: 100% MeOH from 0 to 10 min, followed by an isocratic elution for 10 min with 50% of MeOH and 50% of ProHex. To obtain a standard curve, diolein (0.25 μg/μL) was dissolved in ProHex and quantified.

Likewise, for analysis of ceramide content, lipids from 50 mg liver samples were extracted separately, and the organic phase was hydrolysed in 1 M KOH at 90°C for 60 min, followed by derivatization as previously described (Jani & Ceddia et al., 2023). The samples were then run through a porous stationary‐phased column (Raptor ARC‐18 1.8 μm, 100 mm × 2.1 mm), with chromatography conducted using heptane–isopropyl ether–acetic acid (60:40:3 v/v/v) at a gradient from 0 to 10% in 10 min at a flow rate of 0.8 mL/min, followed by isocratic elution with acetonitrile–deionized distilled water (90:10, v/v) at a flow rate of 1 mL/min. The calibration curve was prepared using *N*‐acetyl‐d‐sphingosine as a standard.

### 2.10 Protein kinase C signalling

To assess membrane and cytoplasmic PKCε expression, membrane and cytosolic fractions were first prepared from 50 mg of frozen liver tissues using the Mem‐PER Plus Membrane Protein Extraction Kit (Thermo Fisher Scientific). Subsequently, liver membrane and cytosolic fractions were lysed in radioimmunoprecipitation assay (RIPA) buffer with protease and phosphatase inhibitors (Sigma). Next, the protein extracts were separated by SDS‐PAGE and transferred to a PVDF membrane. The membrane was incubated overnight at 4°C with antibodies against PKCε (1:1000, Cell Signaling, catalogue number 2683S), Na^+^,K^+^‐ATPase (1:1000, Cell Signaling, catalogue number 3010S) and heat shock protein 90 (HSP90; 1:2000, BD Biosciences, catalogue no. 610418). The Na^+^,K^+^‐ATPase antibody was used as a cell membrane marker, whereas HSP90 was used as a cytoplasmic marker.

To evaluate the expression of PKCζ, whole‐liver protein lysate underwent the already described immunoblotting protocol. However, this time, the membrane was incubated overnight at 4°C with antibodies targeting PKCζ (1:1000 dilution, Cell Signaling, catalogue number C24E6) and glyceraldehyde‐3‐phosphate dehydrogenase (GAPDH; 1:1000 dilution, Cell Signaling, catalogue number 14C10).

Band intensities were quantified using ImageJ software, and PKCε membrane translocation was expressed as the ratio of membrane protein band density to cytosol protein band density, whereas PKCζ protein expression was normalized to GAPDH.

### 2.11 RNA extraction and real‐time PCR

Total RNA from frozen liver was extracted with TRIzol reagent (Invitrogen). Complementary DNA (cDNA) was synthesized from 2 μg total RNA using the High‐Capacity cDNA Reverse Transcription Kit (Invitrogen). Real‐time PCR was performed with the CFX Connect Real‐Time PCR machine (Bio‐Rad Laboratories) using custom‐designed SYBR Green primer sequences included in Table 2. The relative amount of each mRNA was calculated after normalizing to their corresponding β‐actin mRNA, and the results were expressed as a fold change relative to the control group.

**TABLE 2:** Primer sequences for quantitative PCR analysis.

Gene	Forward	Reverse
*Tnfα*	CCCTCACACTCAGATCATCTTCT	GCTACGACGTGGGCTACAG
*Il1β*	GCAACTGTTCCTGAACTCAACT	ATCTTTTGGGGTCCGTCAACT
*Ccl2*	TTAAAAACCTGGATCGGAACCAA	GCATTAGCTTCAGATTTACGGGT
*Ccl3*	TTCTCTGTACCATGACACTCTGC	CGTGGAATCTTCCGGCTGTAG
*Ccl5*	GCTGCTTTGCCTACCTCTCC	TCGAGTGACAAACACGACTGC
*Sptlc1*	ACGAGGCTCCAGCATACCAT	TCAGAACGCTCCTGCAACTTG
*Sptlc2*	AACGGGGAAGTGAGGAACG	CAGCATGGGTGTTTCTTCAAAAG
*Cers1*	CCACCACACACATCTTTCGG	GGAGCAGGTAAGCGCAGTAG
*Cers2*	ATGCTCCAGACCTTGTATGACT	CTGAGGCTTTGGCATAGACAC
*Cers3*	ATGGGCTTGTCTTCGTGAAAG	TTGCTTGTGGAATGCTTGAAAAA
*Cers4*	GCAGACTCAACGCTGGTTCA	TTGCCTTGACCACAGGAACTG
*Cers5*	CGGGGAAAGGTGTCTAAGGAT	GTTCATGCAGTTGGCACCATT
*Cers6*	GATTCATAGCCAAACCATGTGCC	AATGCTCCGAACATCCCAGTC
*Degs1*	GAATGGGTCTACACGGACCAG	CGAGAAGCATCATGGCTACAA
*Degs2*	AGCGACTTCGAGTGGGTCTA	TCCCCGTACTAACCAGCAGG
*Sgms1*	GAAGGAAGTGGTTTACTGGTCAC	GACTCGGTACAGTGGGGGT
*Sgms2*	GAGACAGCAAAACTTGAAGGTCA	CCCGTTGGATAAGGTCTTGGG
*Smpd1*	TGGGACTCCTTTGGATGGG	CGGCGCTATGGCACTGAAT
*Smpd2*	TGGGACATCCCCTACCTGAG	TAGGTGAGCGATAGCCTTTGC
*Smpd3*	TTCTTCGCCAGCCGCTA	CCACCTGCACCTTGAGAAA
*Asah1*	CGTGGACAGAAGATTGCAGAA	TGGTGCCTTTTGAGCCAATAAT
*Asah2*	GCAAAGCGAACCTTCTCCAC	ACTGGTAACAAACAAGAGGGTGA
*Asah3*	TGTGATTCACTGAGGAACTTTCG	AGAAACTTCACTTTTGGCCTGTA
*Acads*	TGGCGACGGTTACACACTG	GTAGGCCAGGTAATCCAAGCC
*Acadm*	GCTGGAGACATTGCCAATCA	GGCGTCCCTCATCAGCTTCT
*Acadl*	TCTTTTCCTCGGAGCATGACA	GACCTCTCTACTCACTTCTCCAG
*Crot*	GAACGGACATTTCAGTACCAGG	CTTCATTTGCGAATGGTTTCACT
*Hadh*	TTGCCAGCAACACGTCTTCTT	GAGGCCAGCAAATCGGTCTT
*Acaa2*	CTGCTACGAGGTGTGTTCATC	AGCTCTGCATGACATTGCCC
*Acat1*	CAGGAAGTAAGATGCCTGGAAC	TTCACCCCCTTGGATGACATT
*Hmgcs2*	GAAGAGAGCGATGCAGGAAAC	GTCCACATATTGGGCTGGAAA
*Hmgcl*	GGTCTCCCCGGCTAAAGTTG	GCCAGAGCTTGACCATAGGTAT
*Bdh1*	TGACACCCGTCGGACCTAC	TTCTCAGTCGGTCACTCTTCA
*Bdh2*	CGACTGGACGGCAAAGTTATT	CCTGGAGTTTGGACTCGTTGA

### 2.12 Statistical analysis

All values are presented as means ± SD. Statistical differences between the means of more than two groups were assessed using one‐way or two‐way ANOVA followed by the Dunnett's or Tukey's multiple comparisons test, respectively. Values of *P* < 0.05 were considered statistically significant. Six to eight mice per group were used for in vivo experiments, and no data were identified outliers by testing or arbitrarily excluded. GraphPad Prism 9 software was used for all data analysis, including the area under the curve analysis for GTTs and PTTs.

## 3 RESULTS

### 3.1 Supplementation with MCTs improves glucose tolerance and insulin sensitivity in obese mice independent of adiposity

**FIGURE 1:**
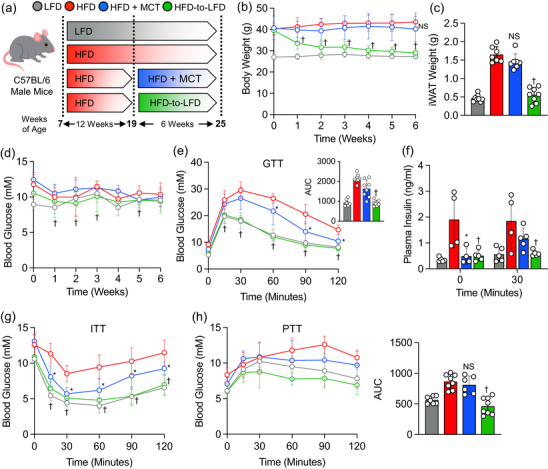
High‐fat diet (HFD) supplemented with medium‐chain triglycerides (MCTs) improves glucose tolerance and insulin sensitivity. (a) Study design. (b–d) Body weight, inguinal white adipose tissue (iWAT) weight and random blood glucose during the 6 week dietary intervention. (e) Glucose tolerance test (GTT) and area under the curve (AUC). (f) Plasma insulin levels during GTT. (g) Insulin tolerance test (ITT). (h) Pyruvate tolerance test (PTT) and AUC. In scatter plots with a bar, one circle represents data from one mouse, and *n* = 6–8 mice per group. Values are presented as means ± SD. Statistical significance was determined using one‐ or two‐way ANOVA. ^*^ *P* < 0.05, HFD + MCTs group versus HFD group. ^†^ *P* < 0.05, HFD‐to‐LFD group versus HFD group.

To assess the potential of supplementation with MCTs, primarily formulated with C8 and C10, in improving dysglycaemia, a 12 week HFD regimen was adapted to induce obesity and insulin resistance in mice. After this, obese mice were assigned randomly to remain on the HFD or transition to HFD + MCTs or LFD for 6 weeks as another dietary intervention model (Figure 1a). A separate cohort of mice was kept on LFD throughout the study and used as a lean control group for comparative purposes. Throughout the 6 week dietary intervention period, obese mice that transitioned to HFD + MCTs did not exhibit changes in body weight, fat mass as evidenced by inguinal white adipose tissue weight or random blood glucose concentrations when compared with their counterparts that remained on an HFD (Figure 1b–d). In contrast, obese mice that transitioned to an LFD showed a marked decrease in body weight, fat mass and random blood glucose concentrations in comparison to those that remained on an HFD (Figure 1b–d). Both dietary interventions, transitioning to either HFD + MCTs or an LFD, significantly improved glucose handling during GTT and its associated insulin levels in comparison to mice that remained on an HFD (Figure 1e,f). Likewise, obese mice that transitioned to HFD + MCTs or an LFD displayed a marked improvement in insulin sensitivity during ITT compared with those that remained on an HFD (Figure 1g). It is worth mentioning that the magnitude of glucose clearance during GTT and ITT was more pronounced in obese mice that were transitioned to an LFD than in those shifted to HFD + MCTs, presumably attributable to the robust decrease in body weight and adiposity observed in the former group (Figure 1b,c). To determine whether enhanced glucose homeostasis was attributed to reductions in hepatic gluconeogenesis, we performed a PTT and found that obese mice that transitioned to an LFD exhibited improvement in glucose excursion in comparison to obese mice that continued on an HFD; however, this improvement was not observed in obese mice that shifted to HFD + MCTs (Figure 1h).

### 3.2 Transitioning to supplementation with MCTs ameliorates hepatic steatosis and inflammation in obese mice

**FIGURE 2:**
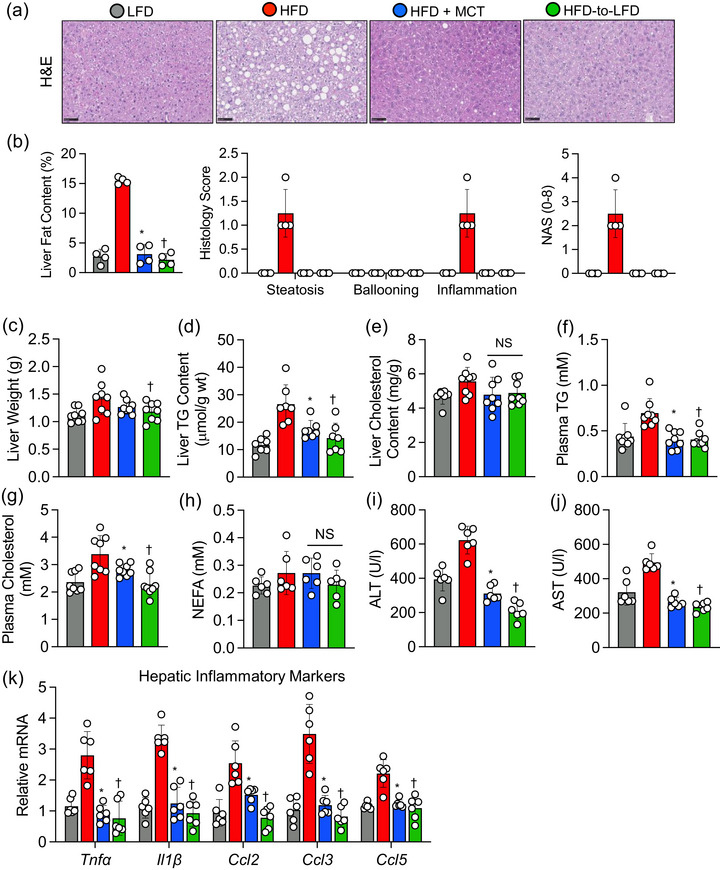
High‐fat diet (HFD) supplemented with medium‐chain triglycerides (MCTs) decreases hepatic steatosis and inflammation. (a, b) Representative images showing Haematoxylin and Eosin (H&E)‐stained liver sections along with histological liver fat quantification and non‐alcoholic fatty liver disease score (NAS). Scale bars: 50 μm. (c) Liver weights. (d, e) Liver triglyceride (TG) and cholesterol content. (f–h) Plasma levels of TG, cholesterol and non‐esterified fatty acids (NEFAs). (i, j) Serum alanine transaminase (ALT) and aspartate transaminase (AST) levels. (k) Hepatic expression of mRNAs encoded by inflammatory‐related genes. In scatter plots with a bar, one circle represents data from one mouse, and *n* = 6–8 mice per group. Values are presented as means ± SD. Statistical significance was determined using one‐way ANOVA. ^*^ *P* < 0.05, HFD + MCTs group versus HFD group. ^†^ *P* < 0.05, HFD‐to‐LFD group versus HFD group. Abbreviations: *Ccl2*, C‐C motif chemokine ligand 2; *Ccl3*, C‐C motif chemokine ligand 3; *Ccl5*, C‐C motif chemokine ligand 5; *Il1β*, interleukin‐1 beta; *Tnfα*, tumor necrosis factor alpha.

Next, we proceeded to explore the potential of supplementation with MCTs in alleviating HFD‐induced hepatic steatosis and inflammation in mice. In contrast to obese mice that remained on an HFD, those that transitioned to HFD + MCTs showed a remarkable decline in hepatic lipid accumulation, as evidenced by Haematoxylin and Eosin staining, to levels similar to those observed in obese mice that transitioned to an LFD (Figure 2a). Consequently, liver histological assessments revealed reductions in steatosis, inflammation and NAS in obese mice that transitioned to HFD + MCTs or an LFD in comparison to their counterparts that remained on an HFD (Figure 2b). Liver weights were significantly reduced in obese mice upon transition to an LFD when compared with those that remained on an HFD (Figure 2c). A discernible trend towards decreased liver weights in obese mice that transitioned to an HFD + MCTs was observed relative to the control obese mice; however, this trend did not attain statistical significance (Figure 2c). Notably, the transition to either HFD + MCTs or LFD resulted in a substantial reduction in hepatic triglyceride levels and in plasma triglyceride and cholesterol levels (Figure 2d–g). There was also a noticeable trend towards reduced hepatic cholesterol levels in the transitioned mice, without any changes in plasma non‐esterified fatty acid levels, in comparison to obese mice that continued on an HFD (Figure 2e,h). Finally, transitioning to either HFD + MCTs or LFD markedly lowered alanine transaminase and aspartate transaminase levels, alongside reducing hepatic mRNA expression of inflammatory genes, such as *Tnfα*, *Il1β*, *Ccl2*, *Ccl3* and *Ccl5*, when compared with obese mice that remained on the HFD (Figure 2i–k).

### 3.3 Supplementation with MCTs lowers hepatic ceramide and diacylglycerol content in obese mice

**FIGURE 3:**
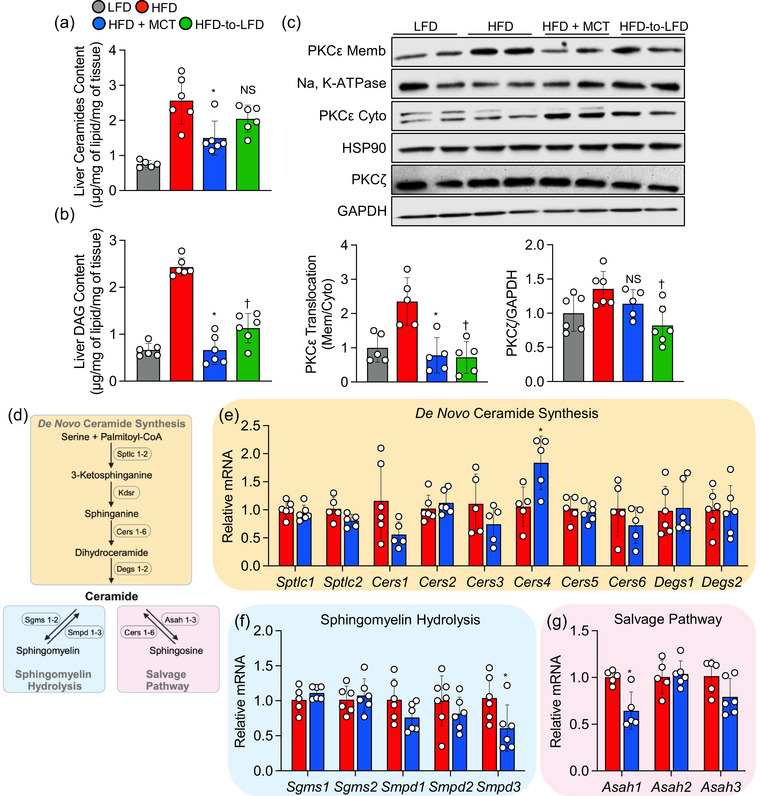
High‐fat diet (HFD) supplemented with medium‐chain triglycerides (MCTs) reduces hepatic diacylglycerol and ceramide content. (a, b) Liver ceramide and diacylglycerol (DAG) content. (c) Representative Western blot bands along with quantification (bottom) showing protein kinase C‐ε (PKCε) protein expression level in membrane (Memb) normalized to Na^+^,K^+^‐ATPase intensity, cytoplasmic (Cyto) normalized to heat shock protein 90 (HSP90) intensity, and whole liver protein kinase C‐ζ (PKCζ) protein expression normalized to GAPDH. (d) Schematic representation of *de novo* ceramide synthesis pathway (light yellow box), sphingomyelin hydrolysis pathway (light blue box) and salvage pathway (light pink box). This schematic diagram was created using BioRender. (e) Hepatic expression of mRNAs encoded by *de novo* ceramide synthesis‐related genes. (f) Hepatic expression of mRNAs encoded by sphingomyelin hydrolysis‐related genes. (g) Hepatic expression of mRNAs encoded by salvage pathway‐related genes. In scatter plots with a bar, one circle represents data from one mouse, and *n* = 6–8 mice per group. Values are presented as means ± SD. Statistical significance was determined using one‐way ANOVA. ^*^ *P* < 0.05, HFD + MCTs group versus HFD group. ^†^ *P* < 0.05, HFD‐to‐LFD group versus HFD group. Abbreviations: *Asah1–3*, acid ceramidase 1–3; *Cers1–6*, ceramide synthase 1–6; *Degs1* and *2*, dihydroceramide desaturase 1 and 2; *Sgms1* and *2*, sphingomyelin synthase 1 and 2; *Smpd1–3*, sphingomyelin phosphodiesterase 1–3; *Sptlc1* and *2*, serine palmitoyltransferase long chain base subunit 1 and 2.

Next, to examine the effects of supplementation with MCTs in reducing hepatic lipotoxicity, we measured liver ceramide and diacylglycerol content as the most studied lipid classes associated with NAFLD (Petersen & Shulman, 2017). As expected, both ceramide and diacylglycerol content were markedly elevated in obese mice maintained on the HFD when compared with mice that were kept on LFD throughout the study (Figure 3a,b). However, after 6 weeks of dietary intervention, obese mice that transitioned to HFD + MCTs demonstrated striking decreases in hepatic ceramide and diacylglycerol levels compared with their obese counterparts maintained on an HFD, whereas obese mice that transitioned to an LFD showed a significant decrease in hepatic diacylglycerol content, but not ceramide levels, compared with obese mice that continued on an HFD (Figure 3a,b). To investigate further the connection between supplementation with MCTs and diacylglycerol metabolism in the context of hepatic steatosis, we measured PKCε translocation to the plasma membrane as an index of PKCε activation. Protein kinase C‐ε is a well‐known sensing kinase that is functionally linked to diacylglycerol accumulation with hepatic insulin resistance (Lyu et al., 2020). Of interest, the transition to either HFD + MCTs or LFD prevented PKCε translocation from the cytosol to the plasma membrane in comparison to obese mice that remained on an HFD (Figure 3c).

Given the well‐established link between ceramide and the activation of PKCζ (Bourbon et al., 2000; Fox et al., 2007), we sought to investigate whether supplementing with MCTs could also mitigate PKCζ activation. Interestingly, transitioning from an HFD to HFD + MCTs resulted in a reduction in whole‐liver PKCζ protein expression in comparison to obese mice that persisted on an HFD; however, this reduction did not reach statistical significance (Figure 3c). In contrast, transitioning from the HFD to LFD significantly decreased hepatic PKCζ protein expression when compared with obese mice that remained on an HFD (Figure 3c).

Given that only obese mice that transitioned to HFD + MCTs exhibited lower hepatic ceramide levels, our focus shifted to ceramide metabolism. We explored gene expression associated with *de novo* ceramide synthesis, sphingomyelin hydrolysis and salvage pathways (Figure 3d). There were no discernible differences in hepatic mRNA levels of serine palmitoyltransferase long chain base subunit 1 and 2 (*Sptlc1* and *Sptlc2*), the rate‐limiting enzymes of *de novo* ceramide synthesis, between obese mice transitioning to HFD + MCTs and those remaining on an HFD (Figure 3e). However, hepatic mRNA levels of ceramide synthase 1, 3 and 6 (*Cers1*, *Cers3* and *Cers6*) in the *de novo* ceramide biosynthesis pathway showed a tendency to decrease in obese mice that transitioned to HFD + MCTs, and there was a concurrent significant increase in *Cers4* mRNA levels in obese mice supplemented with MCTs compared with control obese mice (Figure 3e). Furthermore, among sphingomyelin phosphodiesterase 1–3 (*Smpd1–3*) enzymes catalysing ceramide generation by sphingomyelin hydrolysis, a marked decrease in hepatic mRNA levels of *Smpd3* was observed in obese mice that switched to HFD + MCTs compared with those remaining on an HFD (Figure 3f). Finally, there were significant decreases in hepatic mRNA levels of acid ceramidase (*Asah1*) and a trend towards a decrease in *Asah3*, both responsible for converting sphingosine back to ceramide in the salvage pathway, in obese mice that transitioned to HFD + MCTs compared with their control obese counterparts (Figure 3g). Considering the involvement of *Cers1–6* in the ceramide salvage pathway, it is conceivable that the upregulation of hepatic *Cers4* gene expression, coupled with a significant reduction in *Asah1* gene expression, might represent a compensatory mechanism triggered by the substantial decrease in hepatic ceramide levels observed in the MCTs group.

### 3.4 Transitioning to supplementation with MCTs does not induce nutritional ketosis in obese mice

**FIGURE 4:**
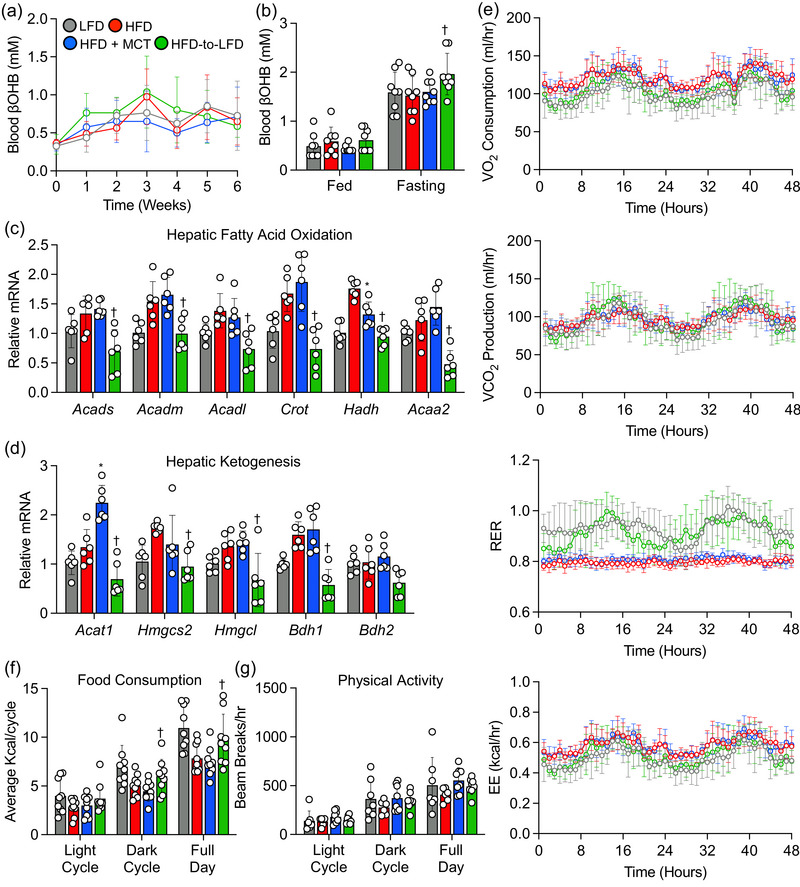
High‐fat diet (HFD) supplemented with medium‐chain triglycerides (MCTs) does not increase hepatic fatty acid oxidation and ketogenesis. (a) β‐Hydroxybutyrate (βOHB) levels during the 6 week dietary intervention. (b) β‐Hydroxybutyrate levels in fed and fasting states. (c, d) Hepatic expression of mRNAs encoded by fatty acid oxidation and ketogenesis‐related genes. (e) Whole‐body oxygen consumption (VO_2_), carbon dioxide production (VCO_2_), respiratory exchange ratio (RER) and energy expenditure (EE) values collected during 48 h. (f, g) Average food consumption and spontaneous activity during 48 h. In scatter plots with a bar, one circle represents data from one mouse, and *n* = 6–8 mice per group. Values are presented as means ± SD. Statistical significance was determined using one‐ or two‐way ANOVA. ^*^ *P* < 0.05, HFD + MCTs group versus HFD group. ^†^ *P* < 0.05, HFD‐to‐LFD group versus HFD group. Abbreviations: *Acaa2*, acetyl‐CoA acyltransferase 2; *Acadl*, acyl‐CoA dehydrogenase long chain; *Acadm*, acyl‐CoA dehydrogenase medium chain; *Acads*, acyl‐CoA dehydrogenase short chain; *Acat1*, acetyl‐CoA acetyltransferase 1; *Bdh1*, 3‐hydroxybutyrate dehydrogenase 1; *Bdh2*, 3‐hydroxybutyrate dehydrogenase 2; *Crot*, carnitine *O*‐octanoyltransferase; *Hadh*, hydroxyacyl‐CoA dehydrogenase; *Hmgcs2*, 3‐hydroxy‐3‐methylglutaryl‐CoA synthase 2; *Hmgcl*, 3‐hydroxymethyl‐3‐methylglutaryl‐CoA lyase.

Although previous studies have indicated that the consumption of MCTs as a nutritional supplement can induce ketosis (St‐Pierre et al., 2019; Vandenberghe et al., 2020), in this study the transition to HFD + MCTs did not increase circulating βOHB in obese mice during both the fed and fasted state when compared with obese mice remained on an HFD (Figure 4a,b). Conversely, the transition to an LFD in obese mice resulted in a significant increase in circulating βOHB levels during the fasting state (Figure 4b). Furthermore, we observed no differences in hepatic mRNA expression of key fatty acid oxidation (*Acads*, *Acadm*, *Acadl*, *Crot* and *Acaa2*) and ketogenesis (*Hmgcs2*, *Hmgcl*, *Bdh1* and *Bdh2*) markers between obese mice continuing on an HFD or transitioning to HFD + MCTs; however, the transition to an LFD significantly lowered mRNA expression of the latter markers (Figure 4c,d). Consistent with this observation, whole‐body oxygen consumption, carbon dioxide production, respiratory exchange ratio and energy expenditure were similar in obese mice that transitioned to HFD + MCTs or remained on an HFD (Figure 4e). Likewise, there were no discernible differences in food consumption and spontaneous physical activity values between obese mice that transitioned to HFD + MCTs or remained on an HFD (Figure 4f,g).

## 4 DISCUSSION

In light of the absence of specific pharmacological remedies for treatment of NAFLD, dietary interventions, including supplementation with MCTs, emerge as a compelling strategy for the treatment of NAFLD. However, owing to variations in the ratio and types of MCT molecules found in nutritional products with MCTs, a controversy has emerged regarding their hepatoprotective effects. In the present study, we showed that supplementation with MCTs, primarily composed of C8 and C10 in a 60/40 ratio, effectively mitigates hepatic steatosis and inflammation while improving liver enzymes and glucose homeostasis in mice subjected to experimental obesity. Our findings are in agreement with previous studies indicating that 10 weeks of dietary intervention with MCTs, comprising 60% C8 and 40% C10, significantly reduces hepatic steatosis and improves glucose homeostasis in both lean and obese mice (Rial et al., 2020). Furthermore, 10 weeks of HFD feeding supplemented with MCTs, consisting of 67% C8 and 23% C10, was sufficient to ameliorate insulin resistance and inflammation in obese mice (Geng et al., 2016). Notably, Goetzman et al. (2020) reported that feeding *Sirt5* knockout mice, which lack a lysine desuccinylase that regulates fatty acid oxidation and ketogenesis, an HFD containing high amounts of C12 found in coconut oil for 5 weeks resulted in periportal macrovesicular steatosis. Interestingly, replacing C12 with MCT oils, containing 60% C8 and 40% C10, for the same duration, did not produce overt macrovesicular steatosis, although a modest degree of microvesicular steatosis was observed in *Sirt5* knockout mice when fed MCT oils. Similar findings were also observed in rats, in which 21 days of supplementation with MCTs led to a dose‐dependent reduction in hepatic steatosis (Ronis et al., 2013). Conversely, rats subjected to HFD feeding for 12 weeks, followed by transitioning to an HFD supplemented with high quantities of C12 for 30 days, exhibited significant increases in hepatic triglyceride content and fat accumulation in the liver (Ströher et al., 2020). Collectively, these studies suggest that supplementation with MCTs rich in C8 and C10 exerts distinct hepatoprotective effects compared with C12 found in coconut oil.

Another important observation in our study is that obese mice, when subjected to dietary intervention with MCTs, displayed a remarkable reduction in hepatic steatosis and inflammation, akin to the levels observed in obese mice that transitioned to an LFD. Importantly, unlike the robust decrease in body weight and adiposity seen in obese mice that transitioned to an LFD, the former hepatoprotective effects observed during the dietary intervention with MCTs did not stem from a reduction in body weight or adiposity, nor were they associated with alterations in food consumption, spontaneous activity or energy expenditure. This finding holds particular significance because, although it is well established that weight loss effectively reduces liver fat accumulation and enhances glycaemic control and insulin sensitivity in NAFLD patients (Younossi et al., 2021), the reality is that only a few individuals manage to achieve the required 10% minimum weight loss (Romero‐Gómez et al., 2017). Furthermore, attaining and sustaining weight loss can be a formidable challenge for most obese individuals (Hall & Kahan, 2018). This is primarily attributable to the fact that it typically necessitates a meticulously crafted plan to achieve successful weight loss outcomes (Middleton et al., 2012; Perri et al., 1988).

Nevertheless, although supplementation with MCTs did not yield significant improvements in lessening adiposity in obese mice, it did lead to notable reductions in intrahepatic triglyceride levels coinciding with improvements in glucose handling and insulin sensitivity in obese mice following the transition to the dietary intervention with MCTs. One plausible explanation for this phenomenon might be attributable to decreases in hepatic ceramide and diacylglycerol content, because the accumulation of these lipid intermediates has been associated with NAFLD and hepatic insulin resistance (Petersen & Shulman, 2017). We speculate that the primary mechanism driving the hepatoprotective effects of MCTs lies in the reduction of hepatic lipotoxicity. This claim is supported by our observation that hepatic ceramide levels decreased exclusively in obese mice that transitioned to the dietary intervention with MCTs, but not in those that switched to an LFD. Moreover, the addition of MCTs to the diet resulted in a decrease in the expression of multiple genes involved in both *de novo* ceramide biosynthesis and sphingomyelin hydrolysis pathways, specifically the downregulation of *Cers6* and *Smpd3*. Notably, the expression of Cers6 is particularly linked to C16:0 ceramide levels and insulin resistance (Mizutani et al., 2005). This correlation is strengthened by the findings indicating that mice deficient in Cers6 not only exhibit reduced C16:0 ceramide levels but are also protected from HFD‐induced obesity and glucose intolerance (Turpin et al., 2014). Additionally, both dietary interventions (supplementation with MCTs and switching to an LFD) reduced hepatic diacylglycerol content. As a result, this prevented PKCε translocation from the cytosol to the plasma membrane, ultimately enhancing insulin sensitivity. Our findings align with previous studies demonstrating that lowering hepatic ceramide and diacylglycerol levels can alleviate hepatic steatosis and insulin resistance (Holland et al., 2007; Lyu et al., 2020; Xia et al., 2015).

It has been postulated that the health benefits of MCTs arise from their unique capability to cross the mitochondrial inner membrane swiftly, without relying on the carnitine shuttle system (Bach & Babayan, 1982). This process leads to the generation of surplus acetyl‐CoA, which is subsequently converted into ketone bodies, thereby leading to favourable downstream metabolic changes. However, the dietary intervention involving MCTs used in this study was largely ineffective at increasing βOHB levels, the most abundant circulating ketone body, in both the fed and fasted state. Additionally, this intervention failed to enhance the expression of key genes associated with fatty acid oxidation and ketogenesis in the liver. However, it is not entirely clear why our findings contrast with those of others, who have observed an elevation in circulating ketone bodies after MCT consumption (Hernandez et al., 2020; Lin et al., 2022; Sternberg et al., 2023). One possible explanation could be that most of these studies used MCT‐based ketogenic diets, which typically contained a very low carbohydrate content (10% of total calorie intake) and a high proportion of fats and MCTs (60% of total calorie intake from MCTs and 10% from other dietary fats). In contrast, our study used an HFD supplemented with MCTs, with 30% of the total calories derived from MCTs and the remaining 30% from other types of fats. Hence, variations in the source and distribution of macronutrients across different MCT‐based diets might account for some of the inconsistencies observed between studies. Nevertheless, it is crucial to emphasize that our study demonstrated that the hepatoprotective effects of MCTs are not contingent upon the induction of ketosis by MCTs.

Given that we did not observe an increase in ketogenesis and ketone bodies in our study, it is conceivable that the health benefits of MCTs might stem from the presence of MCFAs in MCTs rather than from ketones. This suggests that MCTs could play a role in mitigating hepatic steatosis and diminishing liver ceramide and diacylglycerol content through the action of MCFAs. In support of this, a recent study demonstrated that MCFAs effectively suppress lipotoxicity and NASH progression by modulating the immune response through G‐protein‐coupled receptor 84 (GPR84) (Ohue‐Kitano et al., 2023). In this process, MCFAs derived from dietary MCTs act on the GPR84 receptor, blocking macrophage hyperactivation. In addition to serving as signalling molecules themselves, MCFA‐induced intracellular signalling might also lead to an increase in the levels of intracellular second messengers, such as AMP (Takikawa et al., 2013). Debeer et al. (1974) reported that perfusing rat liver with C8, which is abundant in MCT supplements, induces a net decrease in ATP and an increase in AMP content. It is well documented that an elevated AMP/ATP ratio activates AMP‐activated protein kinase (AMPK), and activated AMPK inhibits the activity of enzymes involved in lipid synthesis (Herzig & Shaw, 2018), including those contributing to ceramide production. Nevertheless, whether the observed hepatoprotection and reduction in hepatic ceramide and diacylglycerol levels in our study are a direct consequence of higher intake of MCTs or are merely mediated through MCT‐induced intracellular signalling requires further investigation.

**FIGURE 5:**
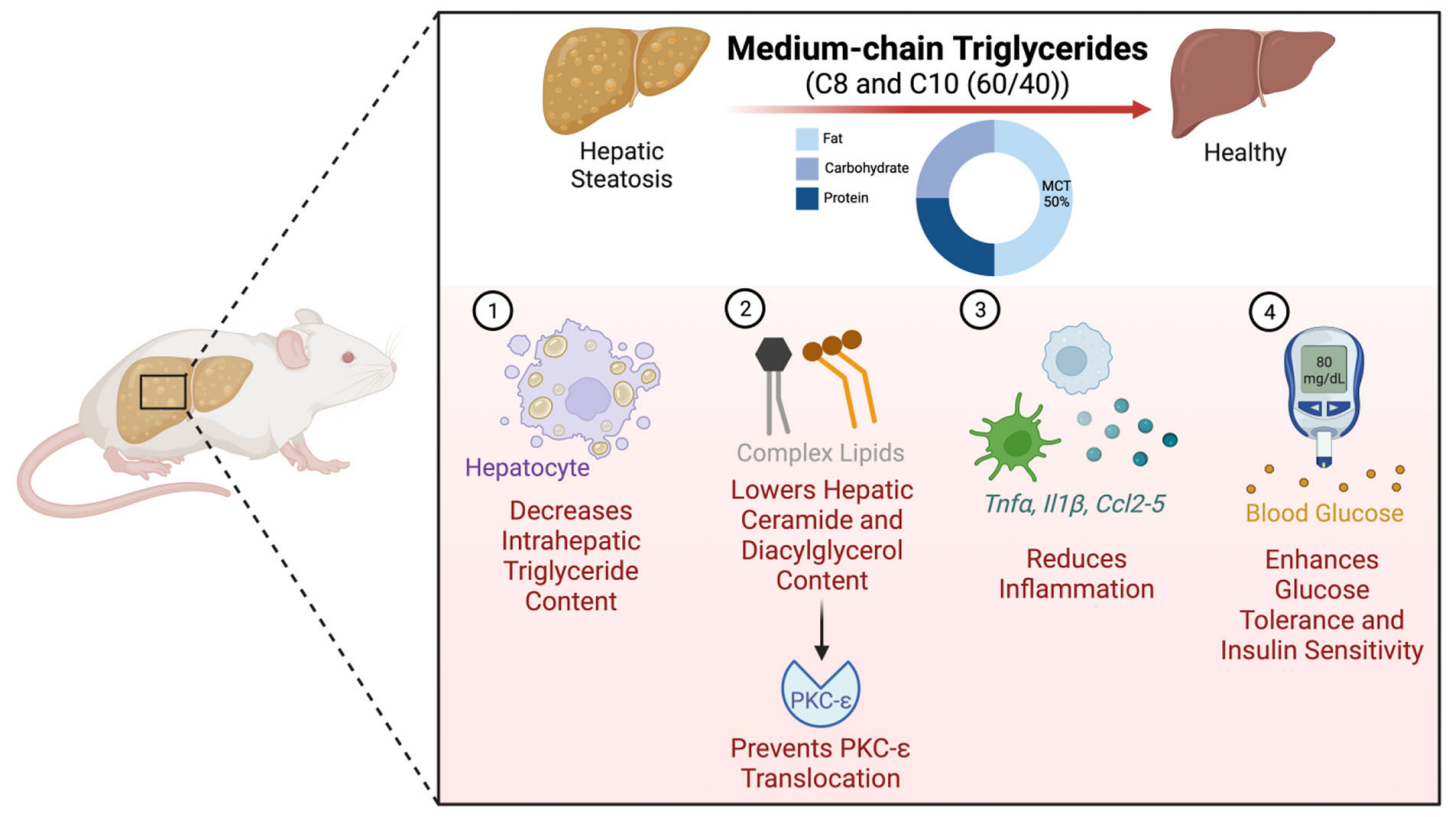
Model depicting the mechanism by which supplementation with medium‐chain triglycerides (MCTs) mitigates hepatic steatosis in obese mice. A high‐fat diet (HFD) supplemented with MCTs, primarily composed of C8 and C10: (1) decreases intrahepatic triglyceride accumulation; (2) lowers hepatic ceramide and diacylglycerol content, which prevents protein kinase C‐ε (PKCε) translocation; (3) reduces hepatic inflammation; and (4) enhances whole‐body glucose tolerance and insulin sensitivity independent of adiposity. This figure was created using BioRender.

In summary, our findings have shown that the supplementation with MCTs, with a predominant composition of C8 and C10, has a significant positive impact on hepatic steatosis and glucose homeostasis (Figure 5). This is achieved through a notable reduction in hepatic ceramide and diacylglycerol content without impacting adiposity. Consequently, in the absence of approved pharmacological remedies for treating NAFLD, the inclusion of MCTs as a dietary supplement emerges as a promising strategy in the ongoing battle against this devastating disease.

## AUTHOR CONTRIBUTIONS

Stephanie Mourad, Abdualrahman M. Abdualkader and Rami Al Batran conceived the project and designed the research. Stephanie Mourad, Abdualrahman M. Abdualkader, Xiaobei Li and Shailee Jani performed the experiments and data analysis. Stephanie Mourad and Rami Al Batran wrote the manuscript. Rolando B. Ceddia and Rami Al Batran reviewed and edited the manuscript.

## CONFLICT OF INTEREST

The authors have no competing interests to disclose.

## Data Availability

The data that support the findings of this study are available from the corresponding author upon reasonable request.
